# Acupuncture for irritable bowel syndrome: A protocol for a pragmatic randomised controlled trial

**DOI:** 10.1186/1471-230X-10-63

**Published:** 2010-06-17

**Authors:** Hugh MacPherson, Martin Bland, Karen Bloor, Helen Cox, David Geddes, Arthur Kang'ombe, Julie Reynolds, Eugena Stamuli, Tracey Stuardi, Helen Tilbrook, David Torgerson, Peter Whorwell

**Affiliations:** 1 Department of Health Sciences, University of York UK; 2 NHS York and North Yorkshire, York, UK; 3 College of Traditional Acupuncture, Hatton, Warwickshire, UK; 4 Wythenshawe Hospital, Manchester, UK

## Abstract

**Background:**

There is insufficient evidence on the effectiveness of acupuncture for irritable bowel syndrome (IBS) for conclusions to be drawn. Given the current interest in acupuncture by patients, it is in the public interest to establish more rigorous evidence. Building on the positive findings from a pilot study, in this paper we present the protocol for a fully-powered trial designed to establish whether or not acupuncture is effective and cost-effective.

**Methods/Design:**

In this pragmatic randomised controlled trial we will randomise patients recruited directly from GP databases to either 10 sessions of acupuncture plus usual GP care or to usual GP care alone. The primary clinical outcome will be the IBS Symptom Severity Score (SSS) (maximum score 500) at three months, and at 12 month assessing whether there is an overall benefit. We estimate the sample size required to detect a minimum clinical difference at 90% power and 5% significance to be 188 patients. To allow for loss to follow up we will recruit 220 patients drawn from an estimated primary care population of 140 000. Analysis will be by intention-to-treat, and multiple imputation is to be used for missing data.

In a nested qualitative study using in-depth interviews, we will explore how patients, acupuncturists, and GPs explain and subsequently understand acupuncture to work. We will use purposive sampling to identify patients and flexible topic guides for the interviews. The data analysis will lead to a thematic description of how patients and practitioners explain how acupuncture works, and whether or not the explanations influence treatment outcome and/or referrals.

We will undertake a cost-effectiveness analysis at 12 months by comparing resource use in the two groups with any treatment benefit. We will use the EQ-5D to measure health-related quality of life and convert into quality adjusted life years (QALYs). We will generate cost effectiveness acceptability curves (CEACs) exploring the probability that acupuncture will produce an acceptable cost per QALY at different cost-effectiveness thresholds.

**Discussion:**

The trial has received NHS ethics approval and recruited 233 patients between November 2008 and June 2009. Results are expected in 2011.

**Trial Registration:**

Current Controlled Trials ISRCTN08827905

## Background

Irritable bowel syndrome (IBS) is the most common disorder encountered by gastroenterologists and the most common functional bowel disorder seen by doctors in primary care [1]. The causes of the disorder are unknown but sufferers are said to experience an increased gastrointestinal stress response consistent with an up-regulation in neural processing between the gut and the brain, termed the "brain-gut axis" [2]. Irritable bowel syndrome is thought to be exacerbated by psychosocial stressors, so treatment is most successful when a multi-component, comprehensive approach is used. Therefore usual care involves a number of interventions as options, including pharmaceuticals, psychological interventions and dietary changes. According to a Cochrane review, bulking agents and antidepressants lack a clear benefit in treating IBS, while antispasmodics, as a category, provide relief of abdominal pain [3]. A review of conventional treatments found that they are rarely effective in managing IBS symptoms [4], a finding that is supported by the Cochrane review [3]. Conventional treatments for IBS are associated with risks including adverse events, worsening of symptoms, and financial costs [5]. The direct and indirect costs associated with IBS are potentially considerable [6]. Some patients are satisfied with current therapies, but many others are frustrated, feeling that advice lacks clarity, conflicts with other GPs' advice and may not work [7].

In this context it is not surprising that patients are increasingly turning to complementary and alternative medicine [8]. An estimated 10% of the UK population uses complementary and alternative medicine [9]. Alternative and complementary techniques are used by between 11% and 43% of patients with gastrointestinal disorders [10]. A survey of GPs found that there is an "effectiveness gap" in currently available treatments in primary care for IBS [11]. Among acupuncture patients, 5% state that their primary complaint is gastrointestinal, most commonly IBS [12]. According to a recent Cochrane review of acupuncture for IBS, the studies to date are of poor quality, heterogeneous in terms of interventions, controls and outcomes, and therefore provide insufficient evidence to determine if acupuncture is an effective treatment for IBS [13]. A recent safety survey of patient reports over a three month period found that acupuncture treatments performed by competent practitioners are rarely associated with adverse events [14].

In this study, we used our pilot study (ISRCTN32823720) as a platform to design a full-scale trial of acupuncture in order to provide robust evidence on acupuncture as a potential referral option in primary care. The primary objective is to establish rigorous evidence of the clinical effectiveness and cost-effectiveness of acupuncture plus usual GP care when compared to usual GP care alone for patients with irritable bowel syndrome (IBS) in primary care. The secondary objective is to perform a qualitative exploration of the experience of patients, GPs and acupuncturists in using acupuncture to treat irritable bowel syndrome.

## Methods/Design

We will conduct a parallel-arm randomised controlled trial to determine the effectiveness and cost-effectiveness of acupuncture plus usual care compared to usual care alone for the treatment of IBS. The rationale for this unblinded pragmatic design is that it will best answer practical questions regarding the clinical and cost implications of offering acupuncture as an additional treatment option within primary care [15]. This trial design will not ascertain the extent that 'placebo' effects contribute to the overall outcome. This design will not be able to ascertain the contribution to the overall effect from expectation, belief and other non-specific factors such as time and attention. In pragmatic terms, even if some of the effect of the treatment process is due to "placebo", acupuncture cannot be delivered without it, and therefore as with all complex interventions, the important question is whether the overall effect is worth paying for. For patients the primary objective is an improvement in outcome and the contribution of a placebo effect to that improvement is immaterial. The pragmatic trial design provides a real-world comparison to be made for a cost-effectiveness analysis and is directly relevant to policy and decision-makers.

Nested within the trial is a qualitative analysis designed to explore how patients, acupuncturists, and GPs explain and subsequently understand acupuncture to work. The qualitative aspect of this study is designed using a phenomenological approach. Phenomenology is the study of how people describe and experience phenomena, which in this case is acupuncture [16]. The design fulfils a basic and summative evaluation purpose, in that the study will contribute to general knowledge and determine usefulness [17].

### Participant recruitment criteria

To recruit participants, we will use the 'database' recruitment method as we did for the pilot [18]. Participants will be identified via the databases of GP practices in the NHS North Yorkshire & York Primary Care Trust. Our inclusion criteria are that participants must be aged 18 or older, with a diagnosis of IBS from their GP. We will exclude participants who do not speak English, who score less than 100 on the IBS Symptom Severity Score (SSS) [19], who have a current diagnosis of haemophilia, hepatitis, HIV, or cancer, who have had major gastrointestinal surgery in the previous six months, who are pregnant, who have a history of psychosis or substance abuse, or who are receiving acupuncture at the time. Participants will be assessed for IBS using the Rome III criteria [20]. In our pilot study, based in Birmingham, 32 eligible participants consented to participate from four GP practices that together had a registered list size of 20,300 [18]. For the proposed research, we estimate that 220 participants are needed (see below for sample size justification). To recruit this number, we will need a total primary care list size of approximately 140,000 patients, equivalent to 14 GP practices with an average of 10,000 patients each. We plan to recruit GP practices in geographically contained groups, such that each group of practices is located near a clinic that provides acupuncture from qualified acupuncturists. These groups are likely to be in the larger centres of population in the region. Based on our pilot, we estimate that recruitment will take 12 months.

### Randomisation and blinding

We will randomise participants to receive either a short course of traditional acupuncture plus usual GP care or usual GP care alone, using simple concealed randomisation, the most robust method of avoiding possible selection bias [21]. The randomisation sequence will be computer generated at the University of York by an independent data manager at the York Trials Unit, and concealed from the researcher who will subsequently inform participants of their allocation. For those participants allocated to acupuncture, the selection of an acupuncture practitioner will be made by appointment availability and convenience for the participant. As this is a pragmatic trial, neither participants nor researchers will be blind to treatment allocation. Whilst randomisation eliminates selection bias, there are other sources of bias we need to avoid. To assess the impact of participant preferences, we propose to ask participants at baseline for their treatment preferences [22]. To avoid bias due to attrition, participants in both groups will be followed up carefully. Participants will also be given £5 as an incentive to complete the final follow-up questionnaire (they will receive the incentive whether or not they actually complete the forms) in order to reduce attrition rates. Incentives such as this have been shown to improve follow-up rates [23].

### Interventions

Acupuncture will be provided by professional acupuncturists who are registered with the British Acupuncture Council, and have at least three years' experience. The acupuncture will be provided at independent clinics that are geographically near the GP practices from which we are recruiting. The participants in this group will be offered up to 10 treatment sessions over a three month period. Acupuncturists will be trained to follow a treatment protocol adapted from the one that was tested by acupuncturists taking part in the pilot trial [18]. The treatment protocol allows sufficient standardisation to assist replication, yet is flexible enough to allow individualised treatments. Adherence to the protocol will be monitored through the use of practitioner logs, which will be collected throughout the trial. In terms of compliance, we expect to obtain similar levels to those achieved in our pilot in which, on average, patients allocated acupuncture took up eight of the ten sessions offered. All patients will remain under the care of their general practitioner and will continue to receive their usual NHS treatment. Both groups will be able seek care elsewhere according to need. We will collect and report information from patients in both groups at three, six, nine and 12 months regarding usual care and treatments they have received.

### Outcome measures

Our primary outcome measure will be the IBS Symptom Severity Score (IBS SSS). This questionnaire is scored from 0 to 500 (< 75 = no IBS, 75-175 = mild case, 175-300 = moderate and 300+ = severe), and has been validated for use in IBS patients [19]. Our secondary outcomes will be the IBS Non-Colonic Symptom Score (which includes lethargy & tiredness, "wind", backache, and other symptoms) [24], the SF-12 to evaluate patients' health related quality of life [25], and the Hospital Anxiety and Depression Scale [26]. The primary outcome for the cost-effectiveness component of the study will be EQ-5D [27]. We will also collect data on medication use, health services used and days lost from work. Along with baseline data, all outcomes will be sought by postal questionnaire at three, six, nine and 12 months, and where this fails, the main outcome measure will be sought by telephone. We will also seek information associated with hospitalisations. Open text questions will be used to gather qualitative data on patient experiences of acupuncture, including adverse events. We will also collect data on safety and treatment processes from log books completed by the acupuncturists. We will use in-depth interviews with flexible topic guides to collect qualitative data.

### Sample size required

The sample size required for a full-scale trial is based on a minimal clinical difference of 50 points on the primary outcome measure the IBS Symptom Severity Score [19]. A residual standard deviation of 90 points was observed in our pilot [18], and taking into account potential sampling bias [28] by using a one-sided 90% confidence interval for the variance, we estimate an adjusted standard deviation to be 105 points. Using this estimate, the sample size required to detect a difference at 90% power and 5% significance level is 188 patients in a two-arm trial. To allow for loss to follow up, of a similar proportion observed in the pilot, our proposed trial requires 220 patients.

For the nested qualitative study, we will collect data from 30 patients, a sample size that is likely to be sufficient to map the diversity of patients in primary care [29]. We will use purposive sampling guided by a matrix based on gender, intervention, preference, and baseline IBS severity as illustrated below in Table 1.

**Table 1:** Qualitative sampling matrix

Baseline IBS Score	Preferred Acupuncture		Preferred Other		Total	
	Male	Female	Male	Female	Male	Female
**Mild - Moderate**	3-4	3-4	3-4	3-4	**6-8**	**6-8**
**Severe**	3-4	3-4	3-4	3-4	**6-8**	**6-8**
**Total**	**6-8**	**6-8**	**6-8**	**6-8**	**12-16**	**12-16**

We will also collect qualitative data from 10 GPs and 10 acupuncturists using convenience samples of practitioners in North Yorkshire, excluding those involved in the trial. It has been suggested that a sample size of 10 is adequate for phenomenological studies because researchers are seeking common features of experience [30].

### Data analysis

We will use intention to treat analysis; all participants will be included in their randomised groups, whether or not they have received their allocated treatment. The primary outcome will be the IBS SSS at three months, using analysis of covariance while adjusting for baseline scores and GP practice, and at 12 months, using multi-level modelling with IBS SSS scores at 3 month intervals. Sensitivity to missing outcomes data will be addressed using multiple imputation. We will also compare between groups the secondary outcomes of IBS non-colonic symptom scores, quality of life (SF-12) and anxiety and depression (HADS). As a secondary analysis, we will explore the interaction between the effect of acupuncture and variables assessed prior to randomisation: participants' belief in acupuncture, expectations that acupuncture will help their IBS (expectation), patient treatment preference and whether patients received their preferred treatment or not. Further secondary analyses will investigate the effects of variation in outcome between acupuncturists.

For the qualitative data, we will transcribe the interviews, identify recurring themes and develop a conceptual framework to apply to the complete data set. Data analysis will be conducted by two researchers working independently to ensure a thorough analysis. Synthesised data will provide a thematic description of how patients and practitioners understand and explain acupuncture to work, and whether or not this explanation influences treatment outcome and/or referrals.

### Cost effectiveness

We will undertake a cost-effectiveness analysis at 12 months by comparing resource use in the two groups with any treatment benefit in an incremental cost-effectiveness ratio. The primary analysis will calculate cost-effectiveness from an NHS perspective because of the potential to influence health policy. However, we will also perform cost-effectiveness calculations from a societal perspective to ensure that this research will not be limited in its usefulness. We will collect data from patients and GPs about patients' resource use, such as visits to their GP, visits to other health providers, both within and outside the NHS and medication use, as well as measuring their days off and whether usual activities are affected by their IBS. We will use EQ-5D to measure health-related quality of life and convert into quality adjusted life years (QALYs), in order to calculate an incremental cost effectiveness ratio (cost per QALY gained), after adjusting for baseline EQ-5D score [31]. To measure presenteeism and absenteeism, we will use the Work Productivity and Activity Impairment instrument (WPAI) and convert the scores into a monetary value [32]. We will also use a net monetary benefit approach to generate cost effectiveness acceptability curves (CEACs) exploring the probability that acupuncture will produce an acceptable cost per QALY at different cost-effectiveness thresholds. This approach accounts directly for uncertainty around the estimates of costs and effects, which is particularly important as this trial is powered on the basis of the clinical effectiveness measure (IBS SSS) rather than the economic outcome measure (EQ-5D). If the intervention is found to be not cost-effective at 12 months, then evaluation over a longer time frame may be considered.

## Discussion

The proposed research received ethics approval from the York Research Ethics Committee (08/H1311/66). The trial recruited 233 patients between November 2008 and June 2009. Written informed consent has been obtained from all participants. Published results are expected in 2011.

## Abbreviations

EQ-5D: EuroQol health utility measure; IBS: irritable bowel syndrome; SSS: Symptom Severity Score; WPAI: Work Productivity and Activity Impairment Instrument

## Competing interests

The authors declare that they have no competing interests.

## Authors' contributions

HM conceived the study and was the lead applicant for funding application as well as the principal investigator for the trial. MB was a co-applicant for funding and is the supervising statistician for the trial, KB is a co-applicant for funding and is the supervising health economist, HC is one of two trial coordinators, DG is Medical Director of the NYYPCT, a GP Principal, and a co-applicant for funding who has advised on trial design and patient recruitment, AK is the trial statistician, JR conducted the pilot study, was a co-applicant for funding and has advised on the full-scale trial, ES is the trials' health economist, TS has designed aspects of the trial, steered the ethics approval process and collected data on treatments provided, HT is a trial co-coordinator, DT was a co-applicant for funding and has advised on trial methodology, PW is a consultant gastroenterologist who was a co-applicant and advised on clinical aspects of the trial and made available the primary outcome measure for the trial. All authors have read and approved the final manuscript.

## Pre-publication history

The pre-publication history for this paper can be accessed here:

http://www.biomedcentral.com/1471-230X/10/63/prepub
